# Relationships between County Health Rankings and child overweight and obesity prevalence: a serial cross-sectional analysis

**DOI:** 10.1186/s12889-016-3091-0

**Published:** 2016-05-14

**Authors:** Karissa Peyer, Greg J. Welk, Lisa Bailey-Davis, Senlin Chen

**Affiliations:** Iowa State University, 283 Forker Building, Ames, IA 50011 USA; Geisinger Health System, 100 N. Academy Ave., MC 44-00, Danville, PA 17822 USA

**Keywords:** Child obesity, County Health Rankings, Social determinants of health

## Abstract

**Background:**

The County Health Rankings (CHR) system provides health rankings for U.S. counties. These factors may have utility for evaluating and predicting health outcomes. This study examined the association between CHR factors and the prevalence of child overweight/obesity (OWOB) in the state of Pennsylvania over 3 years.

**Methods:**

The prevalence of childhood OWOB was obtained for all Pennsylvania school districts for the 2009-10 through 2011-12 school years. Correlational and inferential statistical analyses were used to examine the associations between the prevalence of OWOB in grades K-6 (OWOB1) and 7-12 (OWOB2) and z-score for the overall CHR Health Factors rank, as well as for individual predictive factors (Health Behaviors, Clinical Care, Social and Economic Factors and Physical Environment).

**Results:**

Low to moderate correlations (0.29–0.43) were found between OWOB1 and CHR factors. Weaker and less consistent correlations were found for adolescents. There was a significantly higher prevalence of OWOB in counties with poorer CHR scores.

**Conclusions:**

County-level adult indicators of health are significantly associated with levels of child obesity. Future studies should examine the relationship between CHR and other health outcomes.

## Background

The *County Health Rankings (CHR)* project provides empirically derived health rankings based on health indicators and factors in most U.S. counties. The system was launched in 2004 to facilitate tracking and evaluation of population health issues by county and state health programmers in Wisconsin. Preliminary evaluation supported the utility of the system, [1] so in 2010, the *CHR* expanded outside of Wisconsin to provide rankings within all other U.S. states. The *CHR* now provides a wealth of data for a variety of adult health factors for almost every county in the United States. Factors ranked by the *CHR* project cover a wide range of topics such as education and income, access to healthy foods and medical care, air and water quality, tobacco and alcohol use, and other aspects of health. However, little research has been done to determine which of the health factors measured by the *CHR* have the strongest ties to specific health outcomes (e.g., weight status) [2, 3].

An advantage of the *CHR* database is the inclusion of various social determinants of health (e.g. income, education, and employment status) that are known to influence health outcomes in adults [4–8]. Income disparities have been previously associated with poor health [9, 10], but it is now clear that education and employment status also interact to dictate social status and income. Educational attainment has been linked to mortality and self-reported health, with consistent findings in both men and women and across various racial/ethnic groups [6, 11]. Educational attainment is theorized to impact health through a number of factors and pathways including health knowledge and literacy related to nutrition, exercise, and substance choices; exposure to hazards; and access to health benefits through employment [6]. Direct relationship between employment and health are less established, but one study reported that full-time employment predicts slower declines in self-perceived health and physical functioning over time for both men and women [8]. Unemployment may result in adverse health directly through increased stress or indirectly through loss of income. A landmark study of job loss [12–14] found, among other physiological changes, increases in cholesterol and decreased immune reaction among individuals after job loss. Unemployment has also been shown to lead to an increase of unhealthy behaviors such as alcohol and tobacco use and unhealthy diet [15–17].

The literature clearly demonstrates powerful links between social determinants and health status in adults, but less is known about the impact of social determinants on youth health outcomes. A number of studies have found increased odds of children being overweight or obese if their families experience poverty or food insecurity [18–26]. Associations have also been observed between parental education and child weight status with prevalence of obesity decreasing with increasing levels of maternal education [22, 27–29]. This type of association between parent education and child obesity may be mediated by the effect of education on income since highly educated parents may have resources and incomes to promote healthier lifestyle behaviors in their families. In support of this possibility, Kristiansen et al. [30] found that children whose parents had higher education levels were more likely to participate in sports, walk home from school, consume fruits and vegetables ≥ 5 times per week, and have less screen time and less consumption of soft drinks and sweets.

Perhaps the strongest relationship that has been established between adult factors and child obesity is that of parental weight status. Numerous studies have found increased odds of child overweight if one or both parents is overweight or obese [22, 25, 27, 28, 31–37]. Odds ratios for a child being overweight range from 1.34 to 14.5 depending on the child weight reference criteria, number of overweight parents, and severity of parental overweight. While genetics obviously plays a key role, role modeling and a shared environment also contribute to these associations.

To date, associations between adult factors and child obesity have been examined primarily at the individual level. However, social ecological models recognize that social and environmental features at the community level constrain or enable key lifestyle behaviors related to weight control [38–41]. For example, a previous study demonstrated that positive nutrition and physical activity environments were associated with a lower likelihood of obesity in children [42]. Another study demonstrated that community-level socio-economic deprivation was associated with disparities in child body mass index trajectories [43]. These studies demonstrate effects of county and regional level factors but a gap in the literature is a broader evaluation of these impacts at a state level. The availability of county-level data through the *CHR* begs the question of whether social and environmental factors in the *CHR* may explain disparities in child health outcomes such as obesity prevalence at the group level. Developers of the *CHR* encourage the use of the rankings to support program and policy initiatives at the county and state levels [1]; however, empirical evidence is first needed to confirm the utility of the rankings for explaining variance in health outcomes at these levels.

The purpose of this study was to evaluate relationships between various county-level health factors and the prevalence of child and adolescent overweight and obesity in Pennsylvania. The overall goal was to evaluate the overall utility of the *CHR* indicators; but it was also possible to test several specific hypotheses about social determinants of health. We specifically hypothesized that measures of family income, education and employment, along with measures of adult health behaviors, would be significantly correlated with child and adolescent obesity. Relationships between these county-level health factors were hypothesized to be stronger in older children (7^th^-12^th^ grade) than in younger children (K-6^th^ grade), due to more established lifestyle behaviors and less dependence on home environments.

## Methods

### County Health Rankings

The *CHR* provides rankings for a number of factors within two broad categories: Health Factors and Health Outcomes. The Health Outcomes, which measure life expectancy and quality of life, were not evaluated in the current analysis since our focus was on social determinants of health. The aggregated Health Factors category includes 13 measures under four subcategories. The subcategories include Health Behaviors (tobacco use, diet and exercise, alcohol use, and sexual activity), Clinical Care (access to care and quality of care), Social and Economic Factors (education, employment, income, family and social support, and community safety), and Physical Environment (environmental quality and built environment). The data that inform these measures are gathered from existing surveillance methods such as the National Center for Health Statistics, the Behavioral Risk Factor Surveillance System (BRFSS), the National Center for Chronic Disease Prevention and Health Promotion and many others (See Table 1 for the complete listing of data sources) and are dependent on the data collection window for these respective measures. While the variability in the data collection times for these measures may introduce some error in statistical testing due to children possibly not living in the same location during data collection for all measures, the *CHR* represent a valuable database that bears further evaluation. Rankings and z-scores for all *Ranking* measures are available for public use and download at www.countyhealthrankings.org. Data from 2010, 2011 and 2012 are included in the current analyses. The combined Health Factors z-score and each subcomponent z-score for Health Behaviors, Clinical Care, Social and Economic Factors, and Physical Environment were examined in the current study.

**Table 1:** The *CHR* health factors, focus areas, measures, and sources *CHR*

Health Factor	Focus Area	Measure	Source
Health Behaviors			
	Tobacco Use	Percent of adults that report smoking at least 100 cigarettes and that they currently smoke	BRFSS
	Diet and Exercise	Percent of adults that report a BMI ≥ 30	CDC, National Center for Chronic Disease Prevention and Health Promotion, Division of Diabetes Translation
	Alcohol Use	Motor vehicle deaths per 100 K population; Percent of adults that report binge drinking in the past 30 days	Vital Statistics, NCHS, BRFSS
	Sexual Activity	Chlamydia rate per 100 K population; Teen birth rate per 1,000 female population, ages 15-19	CDC, National Center for Hepatitis, HIV, STD, and TB Prevention, Vital Statistics, NCHS
Clinical Care			
	Access to Care	Percent of population < age 65 without health insurance; Primary care provider rate per 100 K population	Census/Current Population Survey, Small Area Health Insurance Estimates, Health Resources and Services Administration, Area Resource Files
	Quality of Care	Hospitalization rate for ambulatory-care sensitive conditions per 1,000 Medicare enrollees; Percent of diabetic Medicare enrollees that receive HbA1c screening; Percent of chronically ill Medicare enrollees in hospice care in last 6 months of life	Medicare claims/Dartmouth Atlas
Social and Economic Factors			
	Education	Average freshman graduation rate (percent of ninth grade cohort that graduates in 4 years); Percent of population age 25+ with 4-year college degree or higher	National Center for Education Statistics, Decennial Census, American Community Survey
	Employment	Percent of population age 16+ unemployed but seeking work	Local Area Unemployment Statistics, Bureau of Labor Statistics
	Income	Percent of children in poverty; Gini coefficient of income inequality (2010 only, based on household income)	Decennial Census, ACS
	Family and Social Support	Percent of adults without social/emotional support; Percent of all households that are single-parent households	BRFSS, Decennial Census, ACS
	Community Safety	Violent crime rate per 100 K population OR Homicide death rate per 100 K population	Uniform Crime Reporting, Federal Bureau of Investigation, Vital Statistics, NCHS
Physical Environment			
	Environmental Quality	Annual number of unhealthy air quality days due to ozone; Annual number unhealthy air quality days due to fine particulate matter	CDC - Environmental Protection Agency Collaboration
	Built Environment	Percent of zip codes in county with health food outlets (includes grocery stores with >4 employees and produce stands/farmers’ markets); Number of liquor stores per 10 K population (2010 only); Access to recreational facilities (2011 only)	Census Zip Code Business Patterns, Census County Business Patters and Census 2006 Population Estimates

### Body mass index

Pennsylvania Code 28, Chapter 23.7 mandates that height and weight measurements be conducted at least once annually following established procedures, as previously described [44]. Based on the CDC sex-and age-referenced norms, school-calculated body mass index (BMI) data are reported at the school district level including number and percentage of students classified as underweight (below the 5^th^ percentile), normal-weight (from the 5^th^ to the 85^th^ percentile), overweight (from the 85^th^ to the 95^th^ percentile) and obese (at or above the 95^th^ percentile). The overweight (OW) and obese (OB) categories were combined in the current analysis to capture the total number and percentage of OW and OB students (OWOB). The combined indicator is consistent with consensus recommendation to use the 85^th^ percentile as a screening index for overweight [45]. However, analyses were also run with only children at or above the 95^th^ percentile since it is possible that the impact of the county environment on OW would be too subtle to be detected. Analyses were run separately for elementary students in grades K-6 (OWOB1, OB1) and middle school/high school in Grades 7–12 (OWOB2, OB2) to test if effects were stronger in older youth. The serial data collected over time made it possible to examine the stability of the relationships across 3 separate years of BMI data (2009–2010 through 2011–2012) and 3 corresponding years of CHR data (2010 through 2012*).* All school BMI surveillance data were obtained from the Pennsylvania Department of Health and study protocols were approved by the Pennsylvania State University Institutional Review Board.

### Statistical analyses

Descriptive analyses examined the trends in overweight and obesity prevalence in the state of Pennsylvania across 3 years. Repeated measure analyses of variance were used to examine changes in OWOB and OB prevalence and changes in *CHR* over the three successive years of data. Pearson correlations were performed to examine the unadjusted relationships between county-level health factors and childhood overweight/obesity prevalence each year. Multivariate analysis of covariance (MANCOVA) was conducted by entering OWOB1, OWOB2, OB1, and OB2 as outcome variables, overall Health Factors z-score (0 if scores ≤0 or 1 if scores > 0) as the independent variable and year as a covariate.

## Results

There were 500 school districts in Pennsylvania reporting annual body mass index data to the Pennsylvania Department of Health for surveillance purposes. These data are reported as primary (grades K-6) and secondary (grades 7–12) to minimize reporting burden at the school level and to allow comparison with National Health and Nutrition Examination Survey (NHANES) age groups of 6–11 years and 12–19 years, respectively. There are sixty-seven counties in Pennsylvania with a range of 1–43 school districts per county.

The prevalence of overweight and obesity within the state of Pennsylvania did not change significantly from 2009–10 to 2011–12 for either age group (*p* = 0.9746, and 0.6168 for OWOB1 and OWOB2, respectively). Overweight and obesity prevalence were consistently higher in the older age group (33.9–34.5 % across years) than in the younger age group (32.3–32.6 %).

Pearson correlations between county level health indicators and corresponding county levels of overweight/obesity are provided in Table 2. The analyses revealed generally stronger relationships between county-level health factors and overweight prevalence in elementary school children (OWOB1, *r* = 0.39–0.43) than in middle/high school students (OWOB2, *r* = 0.22–0.26). Using Zhu’s absolute thresholds for evaluating correlation coefficients [46], the overall associations with the Health Factor z-score for the younger sample were consistently moderate (average *r* = 0.41) but low for the older sample (average *r* = 0.26). In the younger age group, consistent, significant associations were found for Health Behaviors (average *r* = 0.39), Clinical Care (average *r* = 0.35), and Social and Economic Factors (average *r* = 0.34). Inconsistent and weak correlations were found for the Physical Environment (average *r* = -0.17), with significant associations evident only in 2011–2012. For the older sample, the subscale associations were lower with significance varying across the years (Health Behaviors: average *r* = 0.25, Clinical Care: average *r* = 0.22; Socio-economic Factors: average *r* = 0.25; and Physical Environment: average *r* = -0.25). Correlations were stronger when using OB groups (≥95^th^ percentile) compared to OWOB groups (Table 2).

**Table 2:** Correlations of OWOB and OB Prevalence with *CHR* factor z-scores

	OWOB% K-6th Grade			OWOB% 7-12th Grade		
	Year 1	Year 2	Year 3	Year 1	Year 2	Year 3
Health Factors	0.43	0.42	0.39	0.24	0.19	0.34
	<0.01	<0.01	<0.01	0.05	0.13	<0.01
Health Behaviors	0.40	0.43	0.36	0.17	0.17	0.42
	<0.01	<0.01	<0.01	0.18	0.18	<0.01
Clinical Care	0.29	0.39	0.36	0.20	0.07	0.37
	0.02	<0.01	<0.01	0.10	0.56	<0.01
Social and Economic Factors	0.38	0.30	0.33	0.27	0.23	0.24
	<0.01	0.01	<0.01	0.03	0.06	0.05
Physical Environment	-0.17	0.02	-0.36	-0.22	-0.27	-0.23
	0.16	0.88	<0.01	0.07	0.03	0.0
	OB% K-6th Grade			OB% 7-12th Grade		
	Year 1	Year 2	Year 3	Year 1	Year 2	Year 3
Health Factors	0.65	0.63	0.56	0.28	0.51	0.53
	<0.01	<0.01	<0.01	0.02	<0.01	<0.01
Health Behaviors	0.55	0.59	0.61	0.20	0.42	0.56
	<0.01	<0.01	<0.01	0.20	<0.01	<0.01
Clinical Care	0.46	0.43	0.52	0.22	0.34	0.59
	<0.01	<0.01	<0.01	0.80	<0.01	<0.01
Social and Economic Factors	0.60	0.55	0.50	0.27	0.49	0.43
	<0.01	<0.01	<0.01	0.02	<0.01	<0.01
Physical Environment	-0.22	0.05	-0.44	-0.19	-0.11	-0.44
	0.07	0.71	<0.01	0.12	0.36	<0.01

For each *CHR* factor, pearson correlations are presented with their corresponding p-value below. The Health Factors score represents an aggregate of the four sub-category factors

MANCOVA analyses revealed a significant overall effect between Health Factors z-score and OWOB status in both younger (*F* = 17.64, *p* < 0.0001) and older (*F* = 3.76, *p* = 0.0249) youth. Tests of between-subjects effects showed that the counties in the higher z-score group (zero or above, and therefore a poorer ranking) showed significantly higher OWOB prevalence than those in the lower z-score (below zero, and therefore better ranking), after controlling for year of measurement (Table 3). Parallel analyses limited to only OB status revealed similar but stronger effects with significantly higher OB prevalence in counties with lower z-score in both younger (*F* = 40.76, *p* < 0.0001) and older (*F* = 7.86, *p* = 0.0005) subjects.

**Table 3:** Difference in overweight/obesity prevalence based on factors z-score

	Group	Mean	SD	*F* value	*p*-value
OWOB% K-6th Grade	Lower Z	32.86	3.78	35.27	<0.0001
	Higher Z	35.7	2.96		
OWOB% 7-12th Grade	Lower Z	35.87	6.83	7.28	0.0076
	Higher Z	38.04	4.53		
OB% K-6th Grade	Lower Z	16.77	3.52	40.76	<0.0001
	Higher Z	19.69	2.95		
OB% 7-12th Grade	Lower Z	18.74	6.32	7.86	0.0005
	Higher Z	20.97	5.30		

**Fig. 1:**
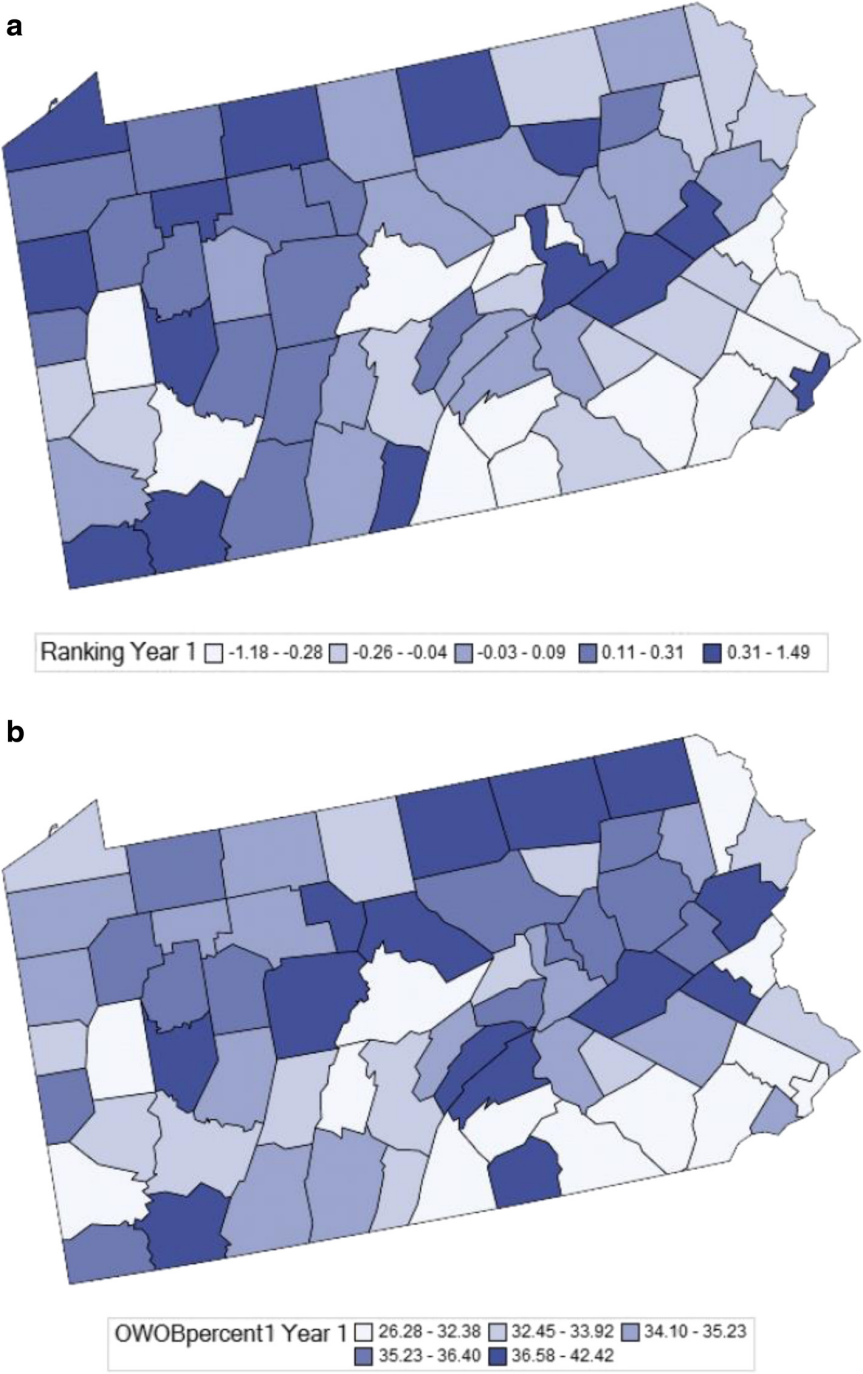
**a**. Distributions of CHR Factor z-score Year 1, 2009-10. **b**. Prevalence of overweight and obesity in elementary school children Year 1, 2009-10

Figure 1a displays the geographic distributions of *CHR* factor rankings across the 67 counties in Pennsylvania for Year 1 of this study. Figure 1b displays the geographic prevalence of OWOB in elementary school children (Grade K-6, OWOB1) in the same year. While there is not perfect agreement between these variables, a distinct pattern can be seen with better *CHR* rankings and lower prevalence of overweight and obesity appearing to cluster in the southeast corner of the state with poorer rankings and higher prevalence of obesity in the northern and western areas of Pennsylvania.

## Discussion

This study provides novel insights about the utility of the *CHR* for research applications. The *CHR* system provides powerful indicators to potentially explain underlying health disparities in children using county health profiles but studies to date have not examined this possibility. The health indicators are based on adult data but it is logical to expect that parenting and home influences would also directly influence youth outcomes.

The present study reveals low to moderate associations were consistently reported between adult Health Factors and youth weight outcomes. These modest correlations are noteworthy since strong correlations cannot be expected between these broad county level indicators. It should also be noted that correlations were generally stronger when analyses were limited to the risk for obesity (≥95^th^ percentile), suggesting a stronger relationship between the *CHR* indicators and the highest level of weight status and health risk.

Individual health is influenced by social and environmental characteristics of the neighborhood or community. It is possible that neighborhood characteristic effects have a larger impact on health through psychological mediators than through social determinants or access to health resources. Ross and Mirowsky found that associations between neighborhood disadvantage and individual health were entirely mediated by perceived neighborhood disorder and resulting fear [47]. These psychological interactions could explain why neighborhood and county level surveillance measures may have weak predictive validity for individual health [48–50]. However, none of these studies examined the relationship between *CHR* and child health so it is difficult to compare our findings to these previous reports.

Previous studies have used other indicators of socioeconomic status (education, employment and income) to examine impacts on child health status. Data from the 1988 National Health Interview Survey Child Health Supplement showed that children from lower income homes were 30 % less likely to report being in excellent health and 80 % more likely to report fair to poor health than children from moderate to higher income homes [51, 52]. These findings are generally consistent with our findings as the Social and Economic Factor had generally consistent (albeit low) associations with obesity across the 3 years. Other studies have found duration of time spent in poverty to be a significant predictor of child health [53]. This relationship cannot be examined in our study since the SES factors in the current study were assessed at the population level and do not capture how long individuals reporting low income or unemployment have been experiencing potential poverty.

An interesting and unexpected observation is that county health factors were more strongly correlated with overweight and obesity in elementary age children than in middle/high school age children. This was contrary to our hypothesis. The stronger association with younger children may reflect the fact that their health is more directly influenced by the lifestyles of their parents. Take for example the Health Behavior category, which includes items capturing exercise, as well as tobacco and alcohol abuse by county. While these indicators are from adults it is possible that these serve as proxy indicators of broader parenting influence or support. Studies have consistently demonstrated positive associations between parent physical activity and child activity so this association is clear. Tobacco use by mothers during pregnancy has been shown to increase odds of obesity in their offspring [54, 55]. While these associations can be defended, the associations may simply reflect better general parenting behaviors. Parents that are active and do not smoke or abuse alcohol may simply be more conscious of their own health and the health of their child. The modest associations (and the differential age effects) clearly merit further exploration.

The Clinical Care indicator was also more consistently related to weight status in the younger sample than in the older sample. This could reflect the likelihood of younger children interacting with a healthcare provider for preventative annual check-ups, a practice that, though recommended, decreases with age [56]. The cross sectional nature of the results preclude any inference of causality but the consistent pattern across the 3 years suggest that broader community/county indicators related to health care may explain variability in youth obesity outcomes. Associations with socio-economic factors were lower but were fairly consistent across the 3 years in both samples. Nau et al. recently demonstrated that socio-economic deprivation may act as a risk-regulator that increases youth exposure to a cluster of environmental factors that promote obesity [43].

A surprising finding was the tendency for some negative correlations between Physical Environment rankings and the prevalence of overweight and obesity. The relationships were not as consistent as with other factors but the negative correlations are hard to explain, as it would be expected that built environment factors would enhance health behaviors and associated outcomes. To examine this, we ran separate correlations to look at the sub factors that make up the environment. These analyses revealed negative associations with Air Quality and a tendency for positive correlations with the Built Environment indicator (data not shown). It is not possible to completely interpret these relationships but it may be due to links with urban areas displaying more positive built environments but poorer air quality or other factors. Among children aged 5–18 years within five regions of Pennsylvania, greater diversity of places for physical activity was associated with lower BMIs but age-dependent variations were observed. For example, higher population density and low county sprawl were associated with lower BMIs among older children only [57]. Younger children may be more dependent on home over community level physical activity features. Additionally, children living in the most rural, least population dense areas had significantly higher rates of obesity compared to urban and suburban populations [44, 58]. Encouraging parents to create home environments that provide support for physical activity among younger and school-age children may be particularly important in the most rural areas, whereas community-level supports may have greater impact for older children and children of all ages in urban areas [58].

It is important to consider other elements that may impact the degree to which *CHR* measures can accurately characterize patterns across a state. Arndt et al. found significant variability across *CHR* focus areas to provide consistent ranking of counties across states [48]. However, they also found a significant, negative correlation between reliability of the measures and number of counties in a state. With 67 counties, Pennsylvania is near the average of the distribution of counties-per-state and therefore may have higher reliability in the *CHR* measures than states with considerably more or less counties.

## Conclusions

In summary, the results revealed low but noteworthy associations between *CHR* indicators and these youth weight outcomes. It is not surprising to see low correlations since there is likely considerable variability within a county in socio-economic factors, environmental resources and clinical care. Individual child and parent factors (e.g., parent weight, low birth weight, rapid infant growth) could be stronger predictors of weight status in children than neighborhood or county level measures. Previous research has consistently shown parent weight status is a strong predictor of child weight, [22, 34–36, 59–61] although it is unclear how much of this relationship is due to genetic factors and how much is due to shared environment. Low birth weight is also a risk factor of adult obesity and diabetes, a correlation that may be intensified by increased weight gain during infancy and enhanced appetite. Differences in maternal care, child birth weight and early child care (e.g. breastfeeding) can also contribute significantly to children’s weight status [62]. Parenting behaviors and environments are also known to influence children’s risk for obesity [63, 64] and these would clearly have a far stronger impact on individual outcomes. Considering the strong impact from genetic and familial influence it is surprising that the associations with *CHR* indicators were as strong as they were. Additional research is clearly needed but these results provide promising insights about the utility of *CHR* indicators for explaining health disparities in the population.

It is important to consider some limitations of the study when interpreting the results. The *CHRs* are aggregated from a number of large data sets that measure health at different levels. Some rankings are based on survey sample (e.g. tobacco use) while others are based on population counts (e. g. teenage births). The efficacy of the rankings could be affected by the measurement method. Additionally, data included in the rankings are from the most recent update of their respective reporting measures (e.g. BRFSS, census, etc.) and so may not necessarily be from the most recent year. For example, the 2010 *CHR* include Vital Statistics data from 2000–2006 and BRFSS data from 2005–2008. This leads to the possibility that the children included in the BMI measurements in the current study were not living in the same location when the health factor data was collected. It also introduces variability in the time course between various health factors and child overweight. It is also important to interpret these results cautiously to avoid drawing conclusions about individuals based on aggregated exposure and outcome variables. Both the *CHR* and BMI data in the current study were reported at the group-level and so results should not be interpreted as explaining any causal relationship between *CHR* variables and weight status in individual children.

Despite the limitations, it is noteworthy that the aggregated *CHR* indicators were significantly associated with parallel county-level indicators of weight status. Future research should work to examine correlations between the *CHR* and other health outcomes. The expanding *CHR* database provides researchers the opportunities to evaluate many research questions across diverse demographic and geographic samples.

## Ethics approval and consent to participate

All study protocols were approved by the Institutional Review Board of Pennsylvania State University.

## Availability of data and materials

Data pertaining to the County Health Rankings is publicly available at www.countyhealthrankings.org.

## Open access

This article is distributed under the terms of the Creative Commons Attribution 4.0 International License (http://creativecommons.org/licenses/by/4.0/), which permits unrestricted use, distribution, and reproduction in any medium, provided you give appropriate credit to the original author(s) and the source, provide a link to the Creative Commons license, and indicate if changes were made. The Creative Commons Public Domain Dedication waiver (http://creativecommons.org/publicdomain/zero/1.0/) applies to the data made available in this article, unless otherwise stated.

## Abbreviations

BMI: body mass index
BRFSS: behavioral risk factor surveillance system
CHR: the County Health Rankings
MANCOVA: multivariate analysis of covariance
NHANES: National Health and Nutrition Examination Survey
OB: obese
OW: overweight
OWOB: overweight and obese
